# ENTRNA: a framework to predict RNA foldability

**DOI:** 10.1186/s12859-019-2948-5

**Published:** 2019-07-03

**Authors:** Congzhe Su, Jeffery D. Weir, Fei Zhang, Hao Yan, Teresa Wu

**Affiliations:** 1 School of Computing, Informatics, Decision Systems Engineering, Arizona State University, Tempe, AZ 85281 USA; 2 Department of Operational Sciences, Graduate School of Engineering and Management, Air Force Institute of Technology, Wright-Patterson AFB, Dayton, OH 45433 USA; 3 Biodesign Center for Molecular Design and Biomimetics, The Biodesign Institute & School of Molecular Sciences, Arizona State University, Tempe, AZ 85281 USA

**Keywords:** Data-driven, Foldability, Sequence segment entropy

## Abstract

**Background:**

RNA molecules play many crucial roles in living systems. The spatial complexity that exists in RNA structures determines their cellular functions. Therefore, understanding RNA folding conformations, in particular, RNA secondary structures, is critical for elucidating biological functions. Existing literature has focused on RNA design as either an RNA structure prediction problem or an RNA inverse folding problem where free energy has played a key role.

**Results:**

In this research, we propose a Positive-Unlabeled data- driven framework termed ENTRNA. Other than free energy and commonly studied sequence and structural features, we propose a new feature, Sequence Segment Entropy (SSE), to measure the diversity of RNA sequences. ENTRNA is trained and cross-validated using 1024 pseudoknot-free RNAs and 1060 pseudoknotted RNAs from the RNASTRAND database respectively. To test the robustness of the ENTRNA, the models are further blind tested on 206 pseudoknot-free and 93 pseudoknotted RNAs from the PDB database. For pseudoknot-free RNAs, ENTRNA has 86.5% sensitivity on the training dataset and 80.6% sensitivity on the testing dataset. For pseudoknotted RNAs, ENTRNA shows 81.5% sensitivity on the training dataset and 71.0% on the testing dataset. To test the applicability of ENTRNA to long structural-complex RNA, we collect 5 laboratory synthetic RNAs ranging from 1618 to 1790 nucleotides. ENTRNA is able to predict the foldability of 4 RNAs.

**Conclusion:**

In this article, we reformulate the RNA design problem as a foldability prediction problem which is to predict the likelihood of the co-existence of a sequence-structure pair. This new construct has the potential for both RNA structure prediction and the inverse folding problem. In addition, this new construct enables us to explore data-driven approaches in RNA research.

## Background

Ribonucleic acid (RNA), as an emerging nanoscale building block, is regarded as one of the most promising candidates to create nano-architectures and nano-devices for therapeutic and diagnostic purposes. Due to its unique biochemical properties and functionalities [1], such as catalysis of metabolic reactions [2], regulation of gene expression [3], and organization of proteins into large machineries [4], RNA has attracted great attention from both academia and industry resulting in broad applications. For example, the success in clinical trials has proved that RNA-based therapeutics hold great potential to overcome the limitation of existing medicine that can only target a limited number of proteins [5]. To fully explore and utilize RNA functions, the cornerstone is to study the multi-levels of complicated RNA structures to include the linear ribonucleotide sequence (primary structure), the 2D fold based on canonical Watson-Crick and wobble base-pairings (secondary structure), the 3D fold (tertiary structure), and the complex spatial arrangement of multiple folded molecules (quaternary structure) [6]. The folding of RNA molecules is broadly considered as a hierarchical process in which the secondary structure will be folded first representing the most relevant characteristic of an RNA molecule [7]. Therefore, studying the RNA secondary structure is one of the fundamental steps towards understanding function-related RNA structures.

In general, RNA secondary structure research falls into two categories: The RNA structure prediction problem, which is to predict the folding result of base pairs given the RNA sequence; and the RNA inverse folding problem, which is to identify the appropriate assignment of nucleotides so that a targeted RNA secondary structure can be folded with certainty. For the RNA structure prediction problem, researchers have developed a variety of computational approaches to increase the prediction accuracy. One early effort is to use the comparative approach to infer a consensus secondary structure by aligning the given sequence with other existing RNA sequences. This requires large collections of RNA sequences for the analysis. A major challenge of this approach is the limited availability of RNA [8]. An alternative is using a thermodynamic model to predict the secondary structure, which is based on the assumption that a structure with smaller free energy tends to be more stable. Therefore, an optimization problem with the objective being to minimize the free energy is constructed to identify the structures with minimum free energy (MFE). A number of research tools have been developed to serve this purpose. One tool is Mfold [9]. It employs a dynamic programming algorithm to predict the RNA secondary structure with MFE. While promising, the prediction accuracy of Mfold is less than satisfactory leading to some research efforts to improve its performance. For example, RNAstructure [8] incorporates the constraints from experimental data to improve the prediction accuracy. Realizing the uncertainties in the folding process, RNAfold [10] provides the estimated probabilities of base pairs. For the RNA inverse folding problem, the objective is to identify the appropriate sequences minimizing the distance metric (e.g., the number of common base pairs) between the structure folded from the designed sequence to the target secondary structure. One of the first tools is RNAinverse [11]. In RNAinverse, a random sequence is generated, changes of the nucleotide assignment are made locally to minimize the dissimilarities between the structures. Apparently, such a local search strategy may be trapped in a local optimum and the designed sequences are highly depended on the initial seed solution. To address this issue, RNA-SSD [12] is proposed to assign initial bases probabilistically attempting to avoid local trapping. incaRNAtion [13] uses global sampling and weighted sampling techniques to avoid the seed bias in local search. In antaRNA [14], ant colony optimization, an efficient bio-inspired optimization algorithm is implemented to expedite the searching process with high accuracy. All of the algorithms reviewed assume the designed sequence will fold into the MFE structure, which will be used to calculate the distance to a target secondary structure.

As noted, previous research in both structure prediction and inverse folding has heavily relied on free energy as the metric to evaluate the stability of RNA structures [9–16]. The hypothesis here is, given an RNA sequence, the secondary structure with the MFE will be the stable structure which it would fold into with highest likelihood and thus is considered “optimum”; and given a structure, the sequence shall be assigned with nucleotides in the way that MFE is achieved. To test the hypothesis, we started by collecting 167 existing pseudoknot-free RNA sequences from the Protein Data Bank (PDB), it is observed that only 53 RNAs (32%) are in MFE secondary structures. This finding indicates MFE alone may not be a sufficient condition in guiding RNA design. In other words, not all existing RNA structures are folded with the energy being MFE. Often, RNA can still be folded at an energy level close to MFE, we call them suboptimal RNAs. As indicated in Laing [6], RNA may have a large number of alternative suboptimum folding which is known as the multi-conformation RNA issue.

Recognizing the limitations from MFE algorithms, some research has proposed to generate a set of possible structures with near-optimal free energy instead of the MFE secondary structure alone. For example, RNAsubopt provides all the secondary structures within *δ* difference from the MFE [28]. However, the number of possible structures grows exponentially with the increment of different *δ*. Others have developed alternative metrics calculated from partition functions to evaluate the accessibility of the possible secondary structures. These include IPknot, Sfold [29], RNAshapes [30] and RNA profiling [31]. However, although efforts in the field have focused on exploring different metrics, researchers have not reached the consensus on which metrics should be broadly adopted.

In this research, we introduce a new concept: RNA foldability. Let the RNA structure prediction problem be considered as sequence → structure*, and the RNA inverse folding problem be considered as the structure → sequence*. Our foldability is defined as *l*(*structure*, *sequence*), which measures the likelihood of the co-existence of the structure – sequence pair. One motivation of developing this new construct is it can be potentially applied to both the structure prediction and inverse folding problems. For example, given a sequence, a number of possible structures could be folded, foldability *l*(*structure*, *sequence*) can be used to identify the structure with high likelihood. For an inverse folding problem, a number of sequencing candidates can be first identified for a targeted structure, again, foldability *l*(*structure*, *sequence*) here can be used to identify the sequence most likely to fold into the structure. A second motivation of this foldability concept is it enables us to explore data-driven approaches to RNA research. By extracting features from both sequence and structure, multi-parametric machine learning models can be developed to obtain the foldability measures. To achieve this, in conjunction with free energy and other commonly used RNA structural design features (e.g., GC content and base pair percentage), we introduce a new metric to evaluate the diversity of RNA sequence segments termed Sequence-Segment entropy (SSE). A Positive-Unlabeled (PU) learning based data driven framework, ENTRNA, is developed using the features to predict RNA foldability. After training on both pseudoknot-free and pseudoknotted RNAs, ENTRNA shows promising accuracy in predicting RNA foldability. Specifically, it successfully identifies 80% pseudoknot-free RNAs and pseudoknotted RNAs can be folded into the desired structures.

There are two main contributions from our proposed ENTRNA. First, RNA design is reformulated as a foldability prediction problem (*l*(*structure*, *sequence*)) which can evaluate the successful rate of a given pair of sequence and structure. This new formulation can fundamentally tackle the challenging issues in RNA design, that is, one RNA sequence may fold into multiple structures, and one RNA structure may have multiple sequence assignments. The second contribution lies in the new metric on assessing the RNA sequence segment diversity. In the remainder of the paper, the ENTRNA is presented in Section 2 followed by validation experiments in Section 3. The conclusion and discussion are drawn in Section 4.

## Methods

### RNA foldability prediction problem

Most existing computational algorithms formulate RNA secondary structure prediction as a deterministic optimization problem which aims to find the global optimal secondary structure for the given sequence. It provides a single best guess for the secondary structure with the assumption that the RNA sequence will only fold into the optimal secondary structure (i.e. MFE secondary structure). Unfortunately, such an assumption has notable limitations as some RNAs (i.e. highly structured ribosomal RNAs) often exist in multiple conformations [17]. Deterministic optimization approaches fail to discover multiple RNA secondary structures.

To address the multi-conformation RNA challenge, we look at RNA design from a different perspective. Specifically, we propose to develop a predictive model to estimate the likelihood *l*(*structure*, *sequence*) of a given RNA sequence folding into a given secondary structure. We call this approach RNA foldability prediction. RNA foldability prediction fundamentally differs from RNA secondary structure prediction and the RNA inverse folding problem, as the later ones only require RNA sequences or secondary structure as a single input. RNA foldability prediction will require both sequence and secondary structure to be provided. As such, it enables foldability evaluation on one sequence vs. its several potential secondary structures. Similarly, it can be used to evaluate one secondary structure vs. its multiple sequence candidates which is the RNA inverse folding problem.

### ENTRNA for RNA foldability prediction

RNA foldability prediction could be regarded as a classification problem. To train a classification model, both successful and failed examples are needed. In the RNA foldability prediction problem, any reported successful synthetic RNA or natural existing RNA can be regarded as a positive example. However, failed RNAs have rarely been reported in the literature. To address this issue, we propose the application of the Positive-Unlabeled Learning technique (PU) to fill in the failed examples. Two different sets of RNA features are defined and extracted for pseudoknot-free and pseudoknotted RNAs respectively. By mapping RNAs into a length-free feature space, it enables us to fully learn and explore all the existing RNAs together. In addition, a new metric is proposed to evaluate the diversity of RNA sequences (see Section 2.2.2). Together with free energy (see Section 2.2.3), base pairing probability (see Section 2.2.4) and other RNA domain knowledge driven features (Section 2.2.5), ENTRNA is developed as a data-driven framework to predict RNA foldability.

#### Generate training dataset for PU learning

PU Learning is originally used to solve the text classification problem, which is to assign predefined labels to a new document [18]. Two datasets are needed for training: a positive labeled training set ***P*** and an unlabeled mixed set ***U***. The positive set ***P*** has the positive examples, the mixed set ***U*** is assumed to have both positive and negative examples, but no explicit class label. Generally, PU learning is a two-step approach. First, it identifies a set of reliable negative examples from the mixed set ***U*** based on the knowledge of positive set ***P***. Next, it builds predictive models on those positive and “negative” examples iteratively and then selects the best model among them.

In the RNA foldability prediction problem, a pair of existing RNA sequence and its corresponding secondary structure is considered a successful example in the positive training set ***P***. The challenge lies in the unlabeled dataset ***U*** as it is not publically available. We decide to generate synthetic RNAs computationally as the examples composing ***U***. The rationale here is the synthetic sequences generated by the computational algorithms are believed to be folded into targeted secondary structures, yet not empirically validated through lab testing, thus could be treated as part of the unlabeled dataset ***U***.

In this research, we use the secondary structures existing in ***P*** as seeds to generate possible sequences. For a given secondary structure in ***P***, instead of randomly assign sequences, we generate a number of possible sequences satisfying three constraints. The first two constraints are the same as in Williams et al. [19]: base pairing and repetition. Base-pairing constraint states only Watson-Crick and G-U base pairs are valid. The repetition constraint sets the longest sequence of bases that can all be the same. For example, if the repetition limit is 4, then AAAA may not appear in the structure, though AAAC can. Given the unique property of RNA folding, the third constraint on GC percentage is added, that is, the minimum and maximum percent of bases in the structure that must be either guanine (G) or cytosine (C). The set of sequences for the given structures consists of our unlabeled dataset ***U***.

Next, we apply PU Learning to identify “reliable” negatives from ***U***. Note we use “reliable” instead of “true” negatives as there is no ground truth to validate the negatives. We make the assumption “reliable” negatives are the ones furthest from the true positives in ***P*** which is known as a prior. For simplicity, we propose to use the Euclidean distance of feature values (see sections 2.2.2–2.2.5 for details on the features) to identify these negatives. Normalization has been done to eliminate the scaling issue of different features. Let ${f}_{u_i,j}$ and ${f}_{p_k,j}^{\prime }$ denote the values of feature j for example ui from **U** and example pk from **P** respectively. ${d}_{u_i}$ is calculated as follows to measure the maximum distance between example *u*_*i*_ to the positive set ***P***:

1 $$ {d}_{u_i}=\max {d}_{u_i,{p}_k}\forall {p}_k\ \epsilon\ P $$

where

2 $$ {d}_{u_i,{p}_k}=\sqrt{\sum_{k=1}^m{\left({f}_{u_i,j}-{f}_{p_k,j}^{\hbox{'}}\ \right)}^2} $$

and *m* is the number of features.

With true positives from ***P*** and “reliable” negatives from ***U***, we are able to develop a classification model (see section 2.2.5) to predict foldability, *l*(*structure*, *sequence*) for any pair of structure - sequence.

#### ENTRNA feature: sequence segment entropy

Due to the incomplete and inaccurate thermodynamic parameters, a great number of RNAs are trapped in the suboptimal structures that are near the predicted global free energy minimum [6]. Meanwhile, the sequence is more likely to be trapped into its suboptimal secondary structures if it has diverse secondary structures. Therefore, a new metric measuring the secondary structure diversity, is needed in addition to free energy.

Entropy, derived from thermodynamics and information theory [20], is used to measure the amount of uncertainty and disorder within a system. Since its inception, entropy has been applied to a diverse set of research fields including structural RNA research. For example, conformational entropy is considered an important factor in protein-ligand discrimination [21]. Positional entropy is introduced to measure the certainty of being unpaired considering all nucleotides [22]. However, the base pairing probability is required for all the existing entropy-based metrics, which is calculated based on the free energy value. Hence, it is still dependent on thermodynamic parameters and it is not capable for pseudoknotted RNAs. Therefore, a pseudoknotted-RNA capable and thermodynamic parameter free metric is needed to evaluate the structural diversity.

The k-mer concept has been widely used in bioinformatics research. For example, in genome, k-mer has been applied to de novo assembly of large genomes from short read sequences [32] and detecting mis-assemblies [33]. In RNA, Sailfish, a k-mer based algorithm, is developed to quantify the abundance of RNA isoforms [34]. In this research, we introduce sequence segment entropy (SSE) to measure the diversity of RNA sequence segments, which is motivated by the k-mer concept. For generalization, assume an RNA sequence of length *n* nucleotides (*nt*_1_, *nt*_2_, …,*nt*_n_), let *w* be the segment size referring to the number of consecutive nucleotides in order. To derive the SSE, we need to evaluate the entire RNA sequence. Thus, we use the moving window concept to list the segments. In that case, the segments of the RNA sequence can be written as:

$$ {\boldsymbol{Seg}}_{\boldsymbol{w}}=\left[{Seg}_w^1,{Seg}_w^2,\dots, {Seg}_w^{n+1-w}\right], $$

where

$$ {Seg}_w^1=\left({nt}_1,{nt}_2,\dots, {nt}_w\right),{Seg}_w^2=\left({nt}_2,{nt}_3,\dots, {nt}_{w+1}\right),{Seg}_w^{n+1-w}=\left({nt}_{n+1-w},{nt}_{n+2-w},\dots {nt}_n\right). $$

Let ***SegU***_***w***_ be the set representing the collection of distinct segments, we have

$$ {\boldsymbol{SegU}}_{\boldsymbol{w}}=\left[{SegU}_w^1,{SegU}_w^2,\dots, {SegU}_w^s\right], where\ s=\left|{\boldsymbol{SegU}}_{\boldsymbol{w}}\right|. $$

Following the entropy calculation, we define *V*_*ent,w*_ as:

3 $$ {V}_{ent,w}=-{\sum}_{i=1}^sp\left({SegU}_w^i\right){\log}_2p\left({SegU}_w^i\right) $$

where

4 $$ p\left({SegU}_w^i\right)=\frac{\#\mathrm{of}\ {SegU}_w^i\  occurence\ in\ {\boldsymbol{Seg}}_{\boldsymbol{w}}}{n+1-w\ }\  for\ i=1,\dots, s $$

Since the value range of SSE is highly dependent on the length of an RNA sequence, we normalize SSE as *RV*_*ent*, *w*_:

5 $$ {RV}_{ent,w}=\frac{V_{ent,w}}{V_{ent,w}^{\ast }} $$

where ${V}_{ent,w}^{\ast }$ is the maximum SSE for segment size w, which is proven to be:

6 $$ {V}_{ent,w}^{\ast }=\left\{\begin{array}{c}-{\log}_2\left(\frac{1}{n+1-w}\right)\  if\ n+1-w\le {4}^w\\ {}-b\ast \frac{a+1}{n+1-w}\ast {\log}_2\left(\frac{a+1}{n+1-w}\right)-\left({4}^w-b\right)\ast \frac{a}{n+1-w}\ast {\log}_2\left(\frac{a}{n+1-w}\right),o/w\end{array}\right. $$

where

$$ a=\left\lfloor \frac{n+1-w}{4^w}\right\rfloor, \kern0.5em b=\left(n+1-w\right)\ \mathit{\operatorname{mod}}\ {4}^w. $$

[**Proposition 1**] Suppose we have two sequences of the same size with probability density set {*p*_1_, *p*_2_, *p*_3_…, *p*_*n* + 1 − *w*_} and {*p*_1_ + *ϵ*, *p*_2_ − *ϵ*, *p*_3_, …, *p*_*n* + 1 − *w*_} and *p*_1_ = *p*_2_ = … = *p*_*n* + 1 − *w*_ = *p* > 0, *ϵ* > 0. The first SSE minus the second SSE equals − *plog*_2_*p* − *plog*_2_*p* + (*p* + *ϵ*) log_2_(*p* + *ϵ*) + (*p* − *ϵ*)log_2_(*p* − *ϵ*)

Since f(x) =  − *xlog*(*x*) is a concave function, according to Jensen’s inequality,

$$ {\displaystyle \begin{array}{l}\frac{1}{2}\ \left(\ \left(p+\epsilon \right){\log}_2\left(p+\epsilon \right)+\left(p-\epsilon \right){\log}_2\left(p-\epsilon \right)\ \right)\\ {}=\frac{1}{2}\ast f\left(p+\epsilon \right)+\frac{1}{2}\ast f\left(p-\epsilon \right)\\ {}<f\left(\frac{1}{2}\ast \left(p+\epsilon \right)+\frac{1}{2}\ast \left(p-\epsilon \right)\right)\\ {}=f(p)=-{plog}_2p\end{array}} $$

Hence, the SSE of the first sequence is greater than the second one. Therefore, the sequence segment should be as uniform as possible to achieve the maximum SSE.

[**Proof on maximum SSE**]*.* The total number of distinct sequence segments with size w is 4^*w*^, since 4 different nucleotides could be assigned to each position arbitrarily. Therefore we have two cases depending on the cardinality of *Seg*_*w*_.

- In the cases where *n* + 1 − *w* ≤ 4^*w*^, the most uniform probability density set will occur when all elements of *Seg*_*w*_ are unique and then each element of *SegU*_*w*_ would have probability $\frac{1}{n+1-w}$.
- In the cases where *n* + 1 − *w* > 4^*w*^ there must exist elements *Seg*_*w*_ that are not unique. The most uniform probability density set will occur when *Seg*_*w*_ is partitioned into two groups of segments. The first group of segments will contain in  *b* = (*n* + 1 − *w*) *mod* 4^*w*^ out of 4^*w*^ and occur more frequently than the remaining group of 4^*w*^ − *b*, which occur in equal amounts. For the group occurring in equal amounts, they must occur exactly $a=\left\lfloor \frac{n+1-w}{4^w}\right\rfloor$ times giving them a probability of $\frac{a}{n+1-w}$. Therefore, the probability for the *b* remaining elements must be $\frac{a+1}{n+1-w}$.

Substituting the optimal probability density sets into Eq. (3), we get Eq. (6).

**[Illustration Example on SSE]** Suppose we have two RNA sequences:

$$ {\displaystyle \begin{array}{l}{\mathbf{seq}}_1=`\mathbf{GAAAAAAAAAAAAAAAAAAC}'\\ {}{\mathbf{seq}}_2=`\mathbf{GACCGUCGUGAGACAGGUUA}'\end{array}} $$

First, we calculate the scaled sequence segment entropy value of **seq**_**1**_, take segment size 3 as an example:

$$ {\displaystyle \begin{array}{l}{\mathbf{Seg}}_3=\left[`\mathrm{GAA}',`\mathrm{AAA}',`\mathrm{AAA}',`\mathrm{AAA}',`\mathrm{AAA}',`\mathrm{AAA}',`\mathrm{AAA}',`\mathrm{AAA}',`\mathrm{AAA}',`\mathrm{AAA}',`\mathrm{AAA}',`\mathrm{AAA}',`\mathrm{AAA}',`\mathrm{AAA}',`\mathrm{AAA}',`\mathrm{AAA}',`\mathrm{AAA}',`\mathrm{AAC}'\right];\\ {}{\mathbf{Seg}\mathbf{U}}_3=\left[`\mathrm{GAA}',`\mathrm{AAA}',`\mathrm{AAC}'\right];\\ {}\mathrm{P}\left(\hbox{'}\mathrm{GAA}\hbox{'}\right)=\frac{1}{18}=0.056;\mathrm{P}\left(\hbox{'}\mathrm{AAA}\hbox{'}\right)=\frac{16}{18}=0.889;\mathrm{P}\left(\hbox{'}\mathrm{AAC}\hbox{'}\right)=\frac{1}{18}=0.056;\\ {}{\mathrm{V}}_{\mathrm{ent},3}=-\left(\frac{1}{18}{\ast \log}_2\frac{1}{18}+\frac{16}{18}{\ast \log}_2\frac{16}{18}+\frac{1}{18}{\ast \log}_2\frac{1}{18}\right)=0.614;\\ {}\mathrm{a}=\mathcal{b}\frac{\left(20+1-3\right)}{4^3}\ \mathcal{c}=0;\\ {}\mathrm{b}=\left(20+1-3\right) \operatorname {mod}\ {4}^3=18;\\ {}{\mathrm{V}}_{\mathrm{ent},3}^{\ast }=-{\log}_2\frac{1}{18}=4.170;\\ {}{\mathrm{RV}}_{\mathrm{ent},3}=\frac{0.614}{4.170}=0.147;\end{array}} $$

Following the same steps above, we get RV_ent, 3_ of **seq**_**2**_ is 0.947. The second sequence (**seq**_**2**_) may fold into more possible structures than the first one. This is reflected by scaled segment entropy value. The *RV*_*ent*, 3_ of first sequence is 0.147, while the value of second sequence is 0.947. The higher scaled segment entropy value means the lower certainty of base pairings between RNA segments.

As the segment size increases, SSE converges to 1. To determine the appropriate segment size, we extract 342 RNA sequences from the PDB database and calculate their normalized SSE with different segment sizes starting with 3 and increment by 1. For each SSE calculated, we also calculate a condition index to check the linear dependency. Following Grewal [23], if the condition index is greater than 30, we conclude there exist high linear dependencies among the SSEs (from varied segmentation size). This is the indicator that at least one SSE with a specific segment size can be derived from a linear combination of SSEs from other segment sizes. In that case, adding more SSE would not contribute to distinguishing the RNA sequence. As seen in Table 1, the maximum condition indices reach > 30 when the segment size 9 is added. Therefore, we determine that the segment size should be 3 to 8. As a result, six SSE features are to be derived for the ENTRNA classification model.

**Table 1:** Maximum Condition Index

Segment Size	3–5	3–6	3–7	3–8	3–9
Maximum Condition Index	3.1	6.4	9.8	21.2	35.4

#### ENTRNA feature: free energy

Free energy is used to measure stability of an RNA structure quantitatively. For pseudoknot-free RNAs, both the free energy value (*V*_*fe*_) of a given pair of sequence and structure and the minimum free energy value (*V*_*mfe*_) that the sequence could achieved would be calculated. The program RNAeval [10] of the ViennaRNA − package calculates the free energy value (*V*_*fe*_) of any pair of sequence and secondary structure. We use RNAfold [10] of the ViennaRNA-package to calculate the minimum free energy value so that we could measure the distance between the current structure to the MFE structure in terms of free energy value.

**Fig. 1:**
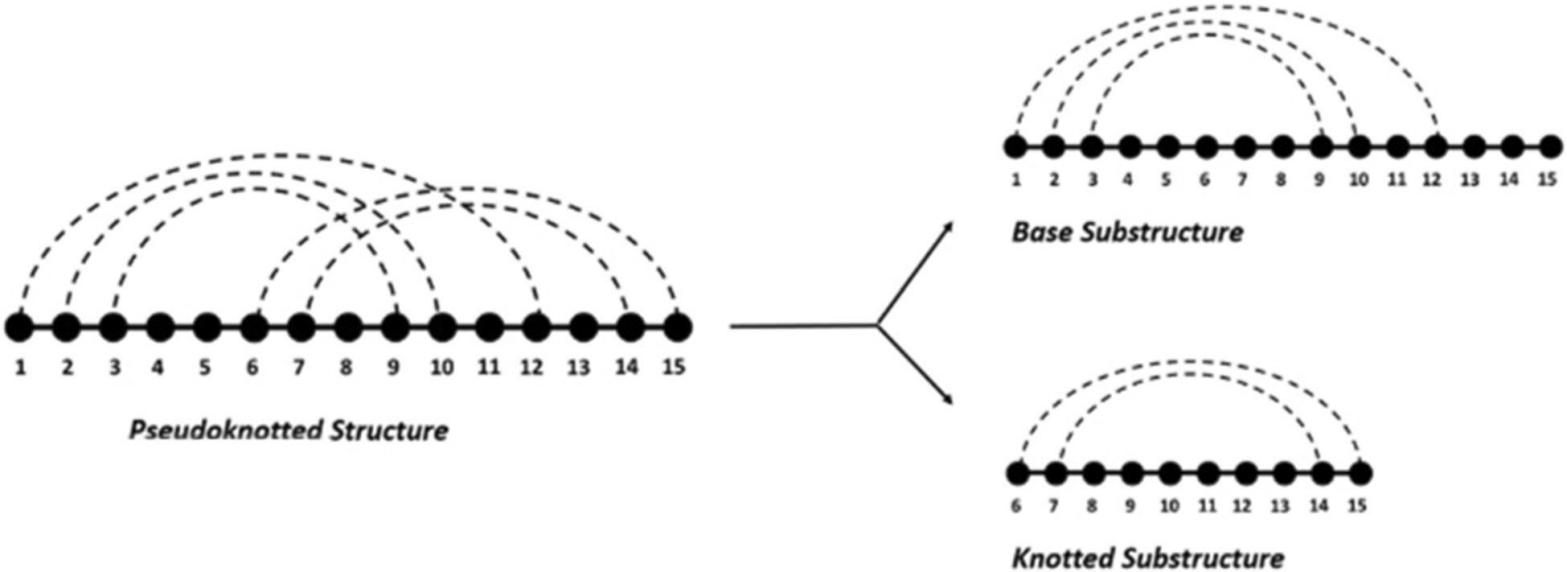
An illustration of the decomposition of a pseudoknotted secondary structure into pseudoknot-free substructures

Unlike the easily computed free energy of pseudoknot-free RNAs, the free energy of pseudoknotted RNA is hard to compute directly due to the inaccurate and incomplete parameters. Inspired by Sato’s idea to decompose pseudoknotted structures into several pseudoknot-free substructures [24], we propose to decompose pseudoknotted structures into a base substructure and knotted substructure(s) (See Fig. 1).

A pseudoknot is typically formed from the base pairings between the unpaired bases in a hairpin loop and those outside the hairpin. Hence, we treat the pseudoknotted structures as the result of two-step folding: First, a pseudoknot-free base substructure is formed as the skeleton structure. Second, the unpaired bases in the hairpin formed by the base substructure form new base pairs with bases outside the hairpin. Specifically, the base substructure is the pseudoknot-free structure that keeps the maximum number of base pairs [25]. It shares the same sequence of the pseudoknotted structure but keeps bases in the knotted substructures unpaired. As a result of further improving structural stability, knotted substructures are formed by keeping the portion of the original sequence that contains additional base pairs that are not knotted. From this viewpoint, it enables the decomposition on arbitrary pseudoknots.

Since both the base substructure and knotted substructures are pseudoknot-free, free energy can be easily calculated. The following free energy based features are extracted for each pseudoknotted RNA by RNAeval [10] and RNAfold [10]:

- *Base substructure free energy* (*V*_*bfe*_): The free energy value given to the sequence and base substructure. It is used to quantitatively measure stability of the base structure;
- *Base substructure minimum free energy* (*V*_*bmfe*_): The minimum free energy value that the sequence could achieve without forming pseudoknots;
- *Knotted substructure free energy* (*V*_*kfe*_): The free energy reduction brought on by the pseudoknots. In addition, we remove the energy increase caused by the “hairpin” since the hairpin is artificially created during the decomposition process.

#### ENTRNA features from base pair probabilities

MFE-based prediction algorithms are generally far from perfect. In general, less than 40% of base pairs could be predicted correctly if a RNA is more than 500 nucleotides [35]. Base pairing uncertainty is considered one of the top reasons. To quantitatively evaluate the base pairing uncertainty, it is assumed that the probability of a secondary structure *s* in equilibrium follows Boltzmann distribution:

6 $$ \mathrm{p}\left(\mathrm{s}\right)\propto {e}^{-E(s)/ RT} $$

where *E*(*s*) is the free energy of the structure, *R* is the gas constant and *T* the thermodynamic temperature of the system. After normalization, the probability of being in secondary structure *s* is:

7 $$ \mathrm{p}\left(\mathrm{s}\right)=\frac{e^{-E(s)/ RT}}{Z} $$

where Z is partition function by summing over all the possible structure:

8 $$ \mathrm{z}={\sum}_s{e}^{-E(s)/ RT} $$

Base pairing probability *p*_*ij*_ is derived by summing up the secondary structure probability with *i* and *j* paired, *q*_*i*_ is the probability of base *i* being unpaired. The following two metrics, calculated by using base pairing probability, have been widely used to evaluate the pseudoknot-free RNA secondary structure uncertainty, which can serve as features in ENTRNA for pseudoknot-free modeling:

- *Ensemble Diversity* (*V*_*ed*_): It measures the expected distance between the target secondary structure and all the other secondary structure. The lower ensemble diversity means the sequence has less ensemble diversity, which further implies the sequence would fold into the target secondary structure with high certainty.
- *Expected Accuracy* (*V*_*ea*_): It measures the expected number of bases that are in correct base pairing status. The higher expected accuracy means more bases are expected to appear in the target secondary structure, which further implies the sequence would fold into the target secondary structure with high certainty.

#### ENTRNA features from RNA domain knowledge

In addition to SSE, free energy and base pairing features, two more features are extracted from domain knowledge:

- *GC Content (Per*_*GC*_): The percentage of guanine or cytosine nucleotides in the sequence. This is a sequence-based feature. GC content is believed to have an impact on RNA stability [26];
- *Base pair percentage (Per*_*bp*_*):* The percentage of base pairs for a given structure. This is a structure-based feature. Base pairs bring free energy reduction in most cases, which influences the structure stability.

In Tables 2, 3 and 4, we summarize all the features including our proposed SSE, free energy, sequence and structural features used for the classification model’s development.

**Table 2:** ENTRNA: Pseudoknot-free and Pseudoknotted RNAs Common Features

Features	Calculation	Description
*Per*_*GC*_	$\frac{\# of\ G\ nts+\# of\ C\ nts\ }{n}$	GC percentage
*Per*_*bp*_	$\frac{\# of\ nts\ that\ are\ base\ paired}{n}$	Base pair percentage
*RV*_*ent*, 3_	$\frac{V_{ent,3}}{V_{ent,3}^{\ast }}$	Normalized SSE with segment size 3
*RV*_*ent*, 4_	$\frac{V_{ent,4}}{V_{ent,4}^{\ast }}$	Normalized SSE with segment size 4
*RV*_*ent*, 5_	$\frac{V_{ent,5}}{V_{ent,5}^{\ast }}$	Normalized SSE with segment size 5
*RV*_*ent*, 6_	$\frac{V_{ent,6}}{V_{ent,6}^{\ast }}$	Normalized SSE with segment size 6
*RV*_*ent*, 7_	$\frac{V_{ent,7}}{V_{ent,7}^{\ast }}$	Normalized SSE with segment size 7
*RV*_*ent*, 8_	$\frac{V_{ent,8}}{V_{ent,8}^{\ast }}$	Normalized SSE with segment size 8

**Table 3:** ENTRNA: Pseudoknot-free RNA Only Features

Features	Calculation	Description
*RV*_*fe*_	$\frac{\left|{V}_{fe}-{V}_{mfe}\right|}{\left|{V}_{mfe}\right|}$	Pseudoknot-free RNA normalized free energy
*V*_*ed*_	$\frac{\sum \limits_{\left(i,j\right)\in s}\left(1-{p}_{ij}\right)+{\sum}_{\left(i,j\right)\notin s}{p}_{ij}\ }{n}$	Ensemble Diversity
*V*_*ea*_	$\frac{\sum \limits_{\left(i,j\right)\in s}2{p}_{ij}+{\sum}_{i\in up}{q}_i\ }{n}$	Expected Accuracy

**Table 4:** ENTRNA: Pseudoknot RNA Only Features

Features	Calculation	Description
*RV*_*bfe*_	$\frac{\left|{V}_{bfe}-{V}_{bmfe}\right|}{\left|{V}_{bmfe}\right|}$	Pseudoknotted RNA base substructure normalized free energy
*RV*_*kfe*_	$\frac{\left|{V}_{bfe}-{V}_{kfe}\right|}{\left|{V}_{bfe}\right|}$	Pseudoknotted RNA knotted substructure normalized free energy
*Per*_*kbp*_	$\frac{\# of\ knot\ base\ pairs}{\# of\ total\ base\ pairs}$	Percentage of knot base pairs

#### Classification model

Based on the training dataset generated, ENTRNA applies logistic regression as a classifier to predict the foldability using 11 features (Tables 2 and 3) for pseudoknot-free and 11 features (Tables 2 and 4) for pseudoknotted RNAs separately. Compared to other classifiers, one advantage of logistic regression is that the result is a continuous value instead of a binary class, which could be explained as the probability of being in the positive class. In this research, the prediction result could be regarded as the foldability for the given pair of sequence and secondary structure. Specifically, we set the foldability threshold as 0.5, which means the given pair of sequence and secondary structure would be classified as a successful case if its foldability value is greater than 0.5. It is our intention to conduct sensitivity analysis on this threshold as one of the future tasks.

## Results

To evaluate the performance of ENTRNA, we measure the model accuracy as the mean of sensitivity and specificity:

$$ Sensitivity=\frac{TP}{TP+ FN} $$

$$ Specificity=\frac{TN}{TN+ FP} $$

where TP is the number of positive examples that are correctly predicted as positive, TN is the number of negative examples correctly predicted as negative, FP is the number of negative examples that are incorrectly predicted as positive and FN is the number of positive examples that are incorrectly predicted as negative.

In order to identify the best feature combinations and parameter settings, we investigate ENTRNA performance exhaustively and record the best parameter settings and feature combinations in terms of Leave-One-Out cross validation accuracy. In addition, a blind test is conducted to evaluate the robustness and generalization of the proposed ENTRNA.

### Dataset

In this research, we prepare 3 separate datasets to train, cross-validate and blind test ENTRNA. The details are as follows:

- Dataset I: 2084 (1024 pseudoknot-free + 1060 pseudoknotted) RNAs from the RNASTRAND database [36]. The length ranges from 4 to 1192 nucleotides. This serves as the training dataset
- Dataset II: 299 (206 pseudoknot-free + 93 pseudoknotted) RNAs extracted by CompaRNA [27] from the PDB database. The length ranges from 20 to 1495 nucleotides. This is used as the test dataset
- Dataset III: 5 laboratory-tested pseudoknotted RNAs with synthetic sequences. All 5 RNA strands were obtained through in vitro transcription and further purified by gel electrophoresis. The RNA strands folded themselves in a buffer solution with a slow cooling process. Among the 5 sequences, 4 of them were not able to produce the designed well-formed rectangle nanostructures. The length of RNA sequences ranges from 1618 to 1790 nucleotides. This is used to test ENTRNA on long structural-complex pseudoknotted RNAs

During the training process, all the RNAs in Dataset I are treated as the positive dataset *P*. To create the unlabeled dataset *U*, we generate 100 sequences for each secondary structure by using existing computational algorithms. Specifically, we use secondary structures in the positive dataset as seed structures, generate the sequence solutions by three different RNA inverse folding algorithms(RNAinverse [11], incaRNAtion [13] and antaRNA [14]). The reason multiple inversion folding algorithms are used is to improve the diversity of the sequence-secondary structure pairs. A pair of seed secondary structure and corresponding sequence defines an example in unlabeled dataset.

### Experiment I: pseudoknot-free RNA

The first experiment is to evaluate ENTRNA on pseudoknot-free RNA. We train and cross-validate the model using 1024 pseudoknot-free RNAs from RNASTRAND to identify the best parameter settings and feature combinations. The model is then blindly tested using 206 RNAs from PDB database. To balance the positive and negative examples, we identify the same number of examples from the unlabeled dataset as “reliable” negative examples. After exhaustively evaluating all the feature combinations, the best performing model, leave-one-out cross validated, is built with the following 5 features:

- Normalized SSE with segment size 3 (RV_ent, 3_)
- GC percentage (Per_gc_)
- Ensemble Diversity (V_ed_)
- Expected Accuracy (V_ea_)
- Pseudoknot-free RNA normalized free energy (RV_fe_)

Since extensive research uses minimum free energy as the single metric to guide RNA design, we provide the MFE result as a reference. Specifically, we implement RNAfold [10] to estimate the MFE structure from the sequence and assess the consistency between the real RNA secondary structure and the MFE predicted RNA secondary structure. If the two structures are identical, the pair of RNA secondary structure and sequence is considered as a positive example under MFE criteria. Table 5 summarizes the comparison between ENTRNA and MFE model on the training and testing datasets.

**Table 5:** Prediction result of ENTRNA on pseudoknot-free RNA

Dataset	ENTRNA Sensitivity	MFE Sensitivity
Training (1024 RNAs from RNASTRAND database)	86.5%	7.4%
Test (206 RNAs from PDB database)	80.6%	25.7%

As observed, in the training and testing, only 76 out of 1024 and 52 out of 206 RNAs are in their MFE secondary structure, which yields the MFE sensitivity to 7.4 and 25.7% separately. In the training procedure, ENTRNA is able to correctly predict 886 pairs of RNA sequence and secondary structure (leave-one-out sensitivity: 86.5%). By directly applying the trained model on the 206 RNAs (blind testing), 165 RNAs are correctly predicted. We conclude ENTRNA model is robust in predicting the foldability of pseudoknot-free RNAs.

### Experiment II: ENTRNA on Pseduoknotted RNA

Following the same procedure as Experiment I, this experiment is to evaluate the performance of ENTRNA on pseudoknotted RNAs. Here we train and leave-one-out cross-validate the model using 1060 pseudoknotted RNAs from RNASTRAND and blindly tested using 93 RNAs from PDB database. The following 3 features are identified in the best performing model:

- Normalized SSE with segment size 3 (RV_ent, 3_)
- Normalized SSE with segment size 8 (RV_ent, 8_)
- Pseudoknotted RNA base substructure normalized free energy (**RV**_**kfe**_)

The free energy calculation for pseudoknotted RNA is still unavailable. Therefore, we only provide the training and test accuracy of ENTRNA, which are summarized in Table 6.

**Table 6:** Prediction result of ENTRNA on pseudoknotted RNA

Dataset	ENTRNA Sensitivity
Training (1060 RNAs from RNASTRAND database)	81.5%
Test (93 RNAs from PDB database)	71.0%

From Table 6, we observe in the leave-one-out cross validated training procedure, ENTRNA is able to correctly predict 864 out of 1060 RNAs (sensitivity: 80.6%). Blind test on the PDB data gives 71.0% sensitivity, that is, 66 out of 93 pseduoknotted RNAs are correctly predicted with foldability. While it is expected blind test will have inferior performance than the training, it is our intention to further explore potential features that could be gathered to improve the predictions.

Next, we validate the model generated from the second experiment blindly on the 5 laboratory long RNA strands. Please note the first two experiments have shown that ENTRNA is able to predict positive examples with high accuracy, while the ability of predicting negative examples could not be validated due to the lack of failed RNAs. Dataset III consists of four failed RNA and one successful RNA which enables us to test the performance of ENTRNA on both sensitivity and specificity. We use the best model trained from Experiment II to predict the foldability of the give RNAs. The model is able to correctly predict the foldability of the one positive example and three out of four negative examples, which yields 100% sensitivity and 75% specificity.

## Discussion

In this paper, we propose a new concept: foldability. It transforms the RNA design problem to a foldability prediction problem - predicting the folding success rate for a given pair of sequence and structure. RNA sequence and secondary structure is a many-to-many mapping, known as multi-conformation. Specifically, each RNA secondary structure could be folded from several RNA sequences and vice versa. In addition, RNA folding is a stochastic process. For each RNA sequence, it will fold into several different secondary structure with certain probabilities. This research proposes a data-driven approach taking the RNA sequence and secondary structure jointly to predict its foldability. The result shows the approach is able to predict RNA foldability with high sensitivity and specificity. This implies the potential promise of the new formulation and its uses in both RNA structure prediction and inverse folding problems.

While successfully, there is room for improvement. First, it is our intention to explore extracting more features to enrich the description of RNA for improved prediction power. Second, we plan to explore the robustness of ENTRNA. One potential issue for all data-driven approaches is the performance is highly dependent on training dataset. In ENTRNA framework, the real world RNAs are not only used in training model, but also identifying reliable negative RNA examples. A larger RNA dataset with both successful and failed (instead of negative) RNA examples will certainly help improve the robustness of the model.

## Conclusion

Introducing thermodynamics (free energy) into RNA folding has been a revolutionary milestone since more than three decades ago. It provides the foundation to computational algorithms for RNA design based on three assumptions: (1) One RNA sequence has a single unique target conformation. (2) The thermodynamic parameters are accurate to derive the free energy characterizing a specific structure. (3) An RNA structure at minimum free energy (MFE) is the most stable structure. The “stable” here refers to the thermodynamic stability calculated in silico. However, recent research has proven that the same RNA sequence may fold into several structures, known as multi-conformation. The thermodynamic parameters used in calculating free energy are only estimates using nearest neighborhood methods. And, many natural RNAs discovered in cells are in an alternative structure with higher-than-the-minimum free energy.

The issues with the three assumptions motivate us to reformulate the RNA structure prediction problem to an RNA foldability prediction problem. As a result, one sequence with its respected multiple potential structures, and one structure with its respected multiple sequences can all be assessed with a unified foldability prediction model. We propose ENTRNA as a data-driven framework for the RNA foldability prediction. In addition, we propose a new metric sequence segment entropy (SSE) as an additional feature for ENTRNA in conjunction with free energy and other RNA domain commonly used features (e.g., GC percentage). Since the unique challenge in designing data-driven approaches for RNA designs is the lack of failure examples, we propose the application of PU (Positive-Unlabeled) learning to make up the failed RNA sequence-structure pairs for the training dataset.

The performance of ENTRNA is validated using both pseudoknot-free and pseudoknotted datasets. In addition, 5 laboratory tested long structural-complex pseudoknotted RNAs with synthetic sequences are used to blindly test the model performance. The superior experiment results show that our method is able to learn from existing RNAs and apply its learning in predicting foldability of unknown RNAs. Unlike previous computational based methods, our method stands at the machine learning perspective to understand and exploit reported RNAs.

## Data Availability

The ENTRNA source code and other necessary resources can be obtained from https://github.com/sucongzhe/ENTRNA.

## Abbreviations

FN: False Negative
FP: False Positive
MFE: Minimum Free Energy
PU: Positive-Unlabeled
SSE: Sequence Segment Entropy
SVM: Support Vector Machine
TN: True Negative
TP: True Positive
